# Prediction of quality of life in early breast cancer upon completion of adjuvant chemotherapy

**DOI:** 10.1038/s41523-021-00296-8

**Published:** 2021-07-13

**Authors:** Alberto Carmona-Bayonas, Caterina Calderón, Raquel Hernández, Ana Fernández Montes, Beatriz Castelo, Laura Ciria-Suarez, Mónica Antoñanzas, Jacobo Rogado, Vilma Pacheco-Barcia, Elena Asensio Martínez, Alejandra Ivars, Francisco Ayala de la Peña, Paula Jimenez-Fonseca

**Affiliations:** 1 Department of Hematology and Medical Oncology, Hospital Universitario Morales Meseguer, University of Murcia, IMIB, Murcia, Spain; 2 Department of Clinical Psychology and Psychobiology, Faculty of Psychology, University of Barcelona, Barcelona, University of Pais Vasco, Pais Vasco, Spain; 3 Department of Medical Oncology, Hospital Universitario de Canarias, Tenerife, Spain; 4 Department of Medical Oncology, Complejo Hospitalario Universitario de Orense, Orense, Spain; 5 Department of Medical Oncology, Hospital Universitario La Paz, Madrid, Spain; 6 Department of Clinical Psychology and Psychobiology, Faculty of Psychology, University of Barcelona, Barcelona, Spain; 7 Department of Medical Oncology, Hospital Universitario Clínico San Carlos, Madrid, Spain; 8 Department of Medical Oncology, Hospital Universitario Infanta Leonor, Madrid, Spain; 9 Department of Medical Oncology, Hospital Central de la Defensa Gomez Ulla, Madrid, Spain; 10 Department of Medical Oncology, Hospital General Universitario de Elche, Elche, Spain; 11 Department of Medical Oncology, Hospital Universitario Central of Asturias, ISPA Oviedo University of Pais Vasco, Pais Vasco, Spain

**Keywords:** Signs and symptoms, Cancer models

## Abstract

Quality of life (QoL) is a complex, ordinal endpoint with multiple conditioning factors. A predictive model of QoL after adjuvant chemotherapy can support decision making or the communication of information about the range of treatment options available. Patients with localized breast cancer (*n* = 219) were prospectively recruited at 17 centers. Participants completed the EORTC QLQ-C30 questionnaire. The primary aim was to predict health status upon completion of adjuvant chemotherapy adjusted for multiple covariates. We developed a Bayesian model with six covariates (chemotherapy regimen, TNM stage, axillary lymph node dissection, perceived risk of recurrence, age, type of surgery, and baseline EORTC scores). This model allows both prediction and causal inference. The patients with mastectomy reported a discrete decline on all QoL scores. The effect of surgery depended on the interaction with age. Women with ages on either end of the range displayed worse scores, especially with mastectomy. The perceived risk of recurrence had a striking effect on health status. In conclusion, we have developed a predictive model of health status in patients with early breast cancer based on the individual’s profile.

## Introduction

In recent decades, we have witnessed an upsurge in the use of patient-reported outcomes (PROs), such as quality of life (QoL), when designing and interpreting clinical trials. These endpoints, together with physician-reported outcomes, are beginning to be taken into account vis-à-vis regulations, as well as therapeutic decision making^1,2^. Thus, the European Society of Medical Oncology Magnitude of Clinical Benefit Scale (ESMO-MCBS), a tool that classifies the magnitude of benefit that can be expected from anticancer treatments, integrates QoL estimations in addition to efficacy and toxicity variables^3^.

In women with breast cancer, QoL has numerous interwoven determinants with mutual, complex, interactions, such as type of surgery, socio-economic status, psychological factors, age, or aspects ranging from body image to fear of recurrence^4,5^. This multiplicity of factors affecting QoL makes this a complex endpoint, which often poses an analytical challenge^1,6,7^. By and large, simplified analyses with bivariate methods, such as Wilcoxon signed-rank tests, on the basis of the variables of interest (e.g., type of surgery) are used to compare QoL. Likewise, in clinical trials longitudinal models are routinely used that focus on time to health-related QoL score deterioration, depending on the study arms^8,9^.

Such univariate analyses have two fundamental drawbacks: (1) their inability to make individual predictions on QoL, conditioned by complex clinical profiles, and (2) the impossibility of pondering interactions a priori deemed relevant^2^. Evidence of these problems is a recent meta-analysis that concluded that modified radical mastectomy (MRM) results in greater decline on several QoL domains than breast-conserving surgery (BCS)^10^. Nevertheless, this methodology is not able to identify the modulating factors of interest, leaving meaningful questions, such as interaction with age, up in the air. Thus, when the inference is made about average subjects, it is not possible to clarify key aspects, for instance, whether seniors experience the same impact on their QoL with a mastectomy as much as younger women^10^. Overall, these limitations impede conveying information regarding the spectrum of treatment options, hinders decision making, and disallows PROs’ support for statements regarding therapeutic effects^7^.

One of the reasons for this imprecision in the literature is that QoL is an ordered categorical variable, which requires the use of specific ordinal regression methods^8,11–13^. The most straightforward is the proportional odds (PO) model^14^, which assumes that the effect of each predictor is similar across all levels of the endpoint^14^. In the real world, it is easy to find examples in which this PO assumption is not met, since the relation with the predictor is not similar for each endpoint category and neighboring values. This conditions the not-so-trivial need to allow for a nonhomogenous effect of a subgroup of predictors across QoL thresholds, treating the variables as nominal, thereby increasing the random error and complexity of analysis^15^. As an intermediate solution, Peterson & Harrell introduced the constrained partial PO (CPPO) model that assumes a linear, monotonic constriction for the coefficients of the variables that diverge from the PO assumption^15,16^. CPPO models are an appealing solution to model QoL based on multiple covariates, by joining accuracy and parsimony. Despite the above, thus far, this method has been uncommon in QoL studies^12,13,17^ or in cohorts of oncological patients^9,18^ given the absence of available software. However, the Coronavirus Disease 2019 (COVID-19) pandemic has prompted the development of a software program, the R rmsb package, that enables Bayesian CPPO models to be simply and efficiently fitted^19^. The reason is that the evolution of viral diseases, similar to QoL, is better captured with ordinal endpoints^20^.

In this situation, the key idea has been to illustrate the complexity, as well as the opportunities that rich modeling of QoL poses by means of this family of ordinal models. Thus, the Bayesian CPPO model is an ideal instrument for both drawing causal inferences and specifying those covariates that predict QoL. As proof of concept with respect to the cross-sectional usefulness of this tool to model these two facets of health status, we have applied these ideas in patients with breast cancer. The primary aim of this study was to predict the health status upon completion of adjuvant chemotherapy adjusted for multiple covariates. Secondary objectives comprised to (1) develop an online calculator to implement this model with potential usefulness for individualized prediction, integrating R with NET; (2) estimate the effect of the type of surgery, based on the individual’s age and profile, and (3) evaluate the incremental benefit of CPPO models versus other alternatives available in this context.

## Results

### Patients

The database comprises 339 patients with breast cancer, 219 of whom were eligible for this analysis, having completed the questionnaires at baseline and upon completion of adjuvant treatment. The recruitment process is shown in Supplementary Fig. 1. The baseline characteristics of the sample are summarized in Table 1, with no substantial differences between participants with or without questionnaires after completing adjuvant chemotherapy (approximately 6 months later). All participants received adjuvant chemotherapy. Sixty-four percent (64.2%) of the HER2-positive and 69.3% of the HER2-negative subjects received postoperative radiotherapy. Supplementary Table 2 displays baseline characteristics based on type of surgery, BCS (*n* = 126, 58%), and total mastectomy (*n* = 93, 42%). In this case, relevant clinical–pathological differences can be seen, with the total mastectomy group presenting a more advanced TNM stage (stage II–III, 78% vs 63%), fewer HER2 + cancers (15% vs 31%), and more ALND (33% vs 16%) than the BSC group. Other sociodemographic characteristics were similar across both groups.

**Table 1:** Patient’s baseline characteristics.

	Overall N = 339 (%)	Patients with questionnaires at the end of adjuvant chemotherapy N = 219 (%)
Age (median, range)	52 (26–81)	53 (28–80)
≤35	15 (4.4)	10 (4.5)
36–45	71 (20.9)	42 (19.1)
46–55	124 (36.5)	79 (36.0)
56–65	75 (22.1)	55 (25.1)
66–75	48 (14.1)	29 (13.2)
75–85	6 (1.7)	4 (1.8)
Sex, male	6 (1.7)	2 (0.9)
ECOG Performance status		
0	276 (81.4)	165 (75.3)
1	59 (17.4)	50 (22.8)
2	4 (1.2)	4 (1.8)
TNM stage		
I	100 (29.5)	66 (30.1)
II	205 (60.5)	130 (59.4)
III	34 (10.0)	23 (10.5)
Menopausal status		
Premenopausal	97 (28.6)	67 (30.5)
Perimenopausal	26 (7.6)	16 (7.3)
Postmenopausal	199 (58.7)	131 (59.8)
Unknown	17 (5.0)	5 (2.2)
HER2-positive cancer	81 (24.0)	53 (24.2)
Estrogen-receptor, positive	213 (62.8)	142 (64.8)
Progesterone-receptor, positive	206 (60.7)	140 (63.9)
Primary tumor surgery		
Breast-conserving surgery	196 (57.8)	126 (57.5)
Mastectomy	143 (42.2)	93 (42.5)
Axillary lymph node dissection	75 (22.1)	52 (23.7)
Adjuvant radiotherapy	226 (66.9)	149 (68%)
Chemotherapy regimen		
Taxane-based	222 (65.5)	152 (69.4)
Anthracycline-based	249 (73.4)	160 (73.0)
Social status		
Secondary or higher education	174 (51.3)	115 (52.5)
Employed	142 (41.9)	100 (45.7)
Married/partnered	258 (76.1)	170 (77.6)
Number of children		
None	67 (19.8)	41 (18.8)
1	68 (20.1)	40 (18.3)
2	150 (44.1)	102 (46.7)
>2	54 (16.0)	36 (16.5)
Number of chronic comorbidities (median range)	0 (0-6)	0 (0-5)
Diabetes mellitus	20 (5.9)	14 (6.3)
Occupational status		
Unemployed, housewife, temporary jobs, or retired	141 (41.5)	103 (47.0)
Unskilled worker	17 (5.0)	10 (4.5)
Semi-skilled worker	5 (1.4)	2 (0.9)
Skilled worker	11 (3.2)	7 (3.2)
Sales	37 (10.9)	26 (11.8)
Administrative-type employment	32 (9.4)	23 (10.5)
Technical or professional	52 (15.3)	40 (18.2)
Business management and/ or public administration	3 (0.8)	3 (1.3)
Not available	41 (12.0)	5 (2.2)

Baseline characteristics were measured before the start of adjuvant chemotherapy

*ECOG* Eastern Cooperative Oncology Group, *TNM* tumor-node-metastases, *HER2* human epidermal growth factor receptor 2.

The most common occupational status was unemployed, housewife, temporary worker, or retired in 41% of the cases. Close to 86% (85.9%) reported that breast cancer had caused them no or very little economic hardship. The distribution of health status is shown in Supplementary Fig. 2, prevailing good or very good scores (e.g., summatory score >50%). Most women perceive risk of recurrence as high and respond very similarly in stages I and II (Supplementary Fig. 3A). The perception of risk of recurrence does not differ on the basis of type of surgery (Supplementary Fig. 3B).

### Effect of surgery on QoL and its domains

**Fig. 1:**
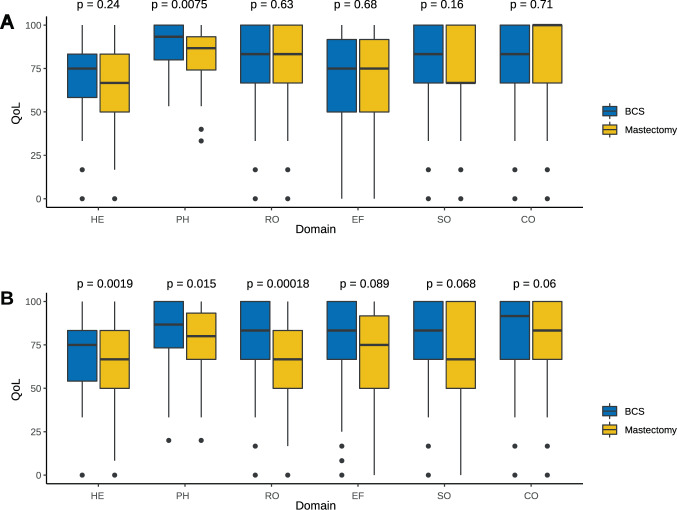
Box plots illustrating the quality-of-life domain scores. (**A**) quality-of-life domain scores pre-chemotherapy; (**A**) and after concluding systemic adjuvant therapy (**B**). Note: The graph presents the median, the first and third quartile (25th and 75th percentiles), maximum scores, and individual points that were outside the extremes of the whiskers. The hormonal status of the six males with breast cancer was coded as unknown. BSC breast-conserving surgery, QoL quality of life, HE health status, PH physical domain, RO role domain, EF emotional functioning, SO social domain, CO cognitive domain.

Figure 1A and B illustrates box plots with the distribution of scores depending on type of surgery. At baseline, the physical status following total mastectomy was only slightly worse compared to BSC, mean 84.6 vs 88.6, respectively (*p* = 0.007). In contrast, upon completion of adjuvant chemotherapy, women who underwent total mastectomy reported a slight increase in symptoms (e.g., mean sum score of 19 vs 24, *p* = 0.002), as well as significant decline on all QoL scores, such as global health status, physical/ role/ emotional functioning, fatigue, and nausea & vomiting (*p* < 0.05) (Supplementary Table 3). For instance, mean global health status scores at the end were 62 vs 70 for total mastectomy vs BCS, respectively (*p* = 0.004).

### Prediction of global health status upon completion of adjuvant chemotherapy

The frequentist PO model for global health status at 6 months is illustrated in Supplementary Fig. 4. With this approach, total mastectomy was associated with worse scores with OR 0.53 (95% confidence interval, 0.27–1.05). The PO assumption was evaluated separately for each of its predictors (Supplementary Fig. 5). The PO holds up well for categorical variables (surgery, perceived risk of recurrence, ALND, chemotherapy, and stage). However, a deviation is seen with respect to quantitative variables (age, EORTC QLQ-C30 sum score), critical if we are to comprehend the individualized effect as a function of them. The multinomial formulation (complete or partial) generates abstruse, implausible models (Supplementary Fig. 6).

To allow departures from the PO, a Peterson–Harrell’s CPPO model was fitted. The coefficients of this more parsimonious model are displayed in Supplementary Fig. 7. The model has an acceptable discriminatory ability, c-index 0.65 (95% highest posterior density interval [HPDI], 0.63–0.67) and a Brier score of 0.21 (95% HPDI 0.19–0.28). Other performance measures are shown in Supplementary Fig. 8.

**Fig. 2:**
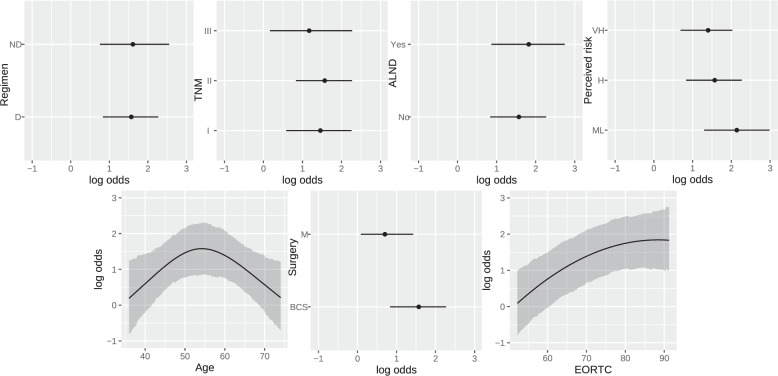
Partial effects on log odds. Partial effect plots are shown with 0.95 highest posterior density intervals. Point estimates are posterior modes. BCS breast-conserving surgery, M mastectomy, ALND axillary lymph node dissection, EORTC European Organisation for Research and Treatment of Cancer, TNM tumor-node-metastases, ML medium-low, H high, VH very high. Note: the EORTC variable refers to the sum score.

**Fig. 3:**
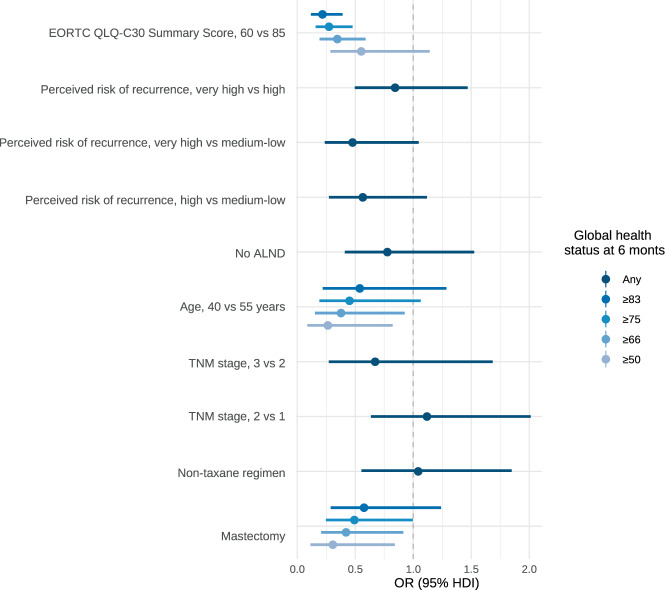
Odds ratios based on the CPPO model. HDI highest density interval, ALND axillary lymph node dissection, TNM tumor-node-metastases, OR odds ratio. Note: odds ratios are based on posterior mode parameters, also displaying the 0.95 highest posterior density intervals. The shades of blue represent different cutoffs of the response variable (health status at 6 months). The plot reveals the linear constrained effect on the predictors. We can see that the CPPO model assumes proportional odds for all variables, except for baseline QoL and age (that interacts with surgery). The model imposes a linear restriction on the coefficients of these variables that do not meet the proportional odds assumption with respect to cutoffs. In contrast, the variables that do meet the proportional odds assumption have a single odds ratio that is valid for the entire range of possible cutoffs.

The analysis of partial effects on the log odds reveals the nonlinear influence of age on global health status (worse on both ends), as well as the risk of worse scores following total mastectomy or with high perceived risk of recurrence (Fig. 2). The consequence of assuming constrained parameters in certain covariates is apparent in the OR plot (Fig. 3). Thus, the magnitude of association between type of surgery and health status gradually decreases if the cutoff increases (e.g., OR 0.30, 0.49, and 0.57, for thresholds of ≥50%, ≥75%, and ≥83%, respectively), denoting a nonhomogenous effect on the spectrum of this domain. This trend is similar when comparing young and middle-aged women. Indeed, the most relevant variables are baseline QoL, extreme age (young or old), and perceived risk of relapse. In a sensitivity analysis, the model was fitted including the tumor stage; this covariate was not associated with QoL, while the other coefficients remained unaltered (data not shown).

**Fig. 4:**
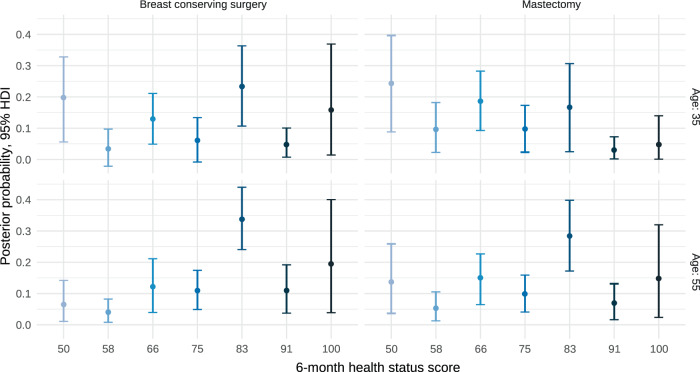
Example of global health status prediction for four patient profiles. The predictions with or in mastectomy in young or middle-aged women are shown. HDI highest density interval.

In the frequentist formulation, neither ALND nor locally advanced stages had a relevant impact after completing adjuvant chemotherapy (Supplementary Fig. 4). Nevertheless, the Bayesian approach estimates 78% and 81% of posterior probability of harm, respectively (Table 2). Likewise, total mastectomy worsened the health status (cutoff ≥ 83) with OR 0.57 (95% HPDI, 0.28–1.23) and this translated as a probability of associated harm of 93%, 84%, and 70% for any magnitude of detriment, effect size >15% and >30%, respectively. Figure 4 illustrates the model’s ability to predict health status across four patient profiles, BSC, or total mastectomy in younger or older women. Finally, we have designed an online calculator (https://www.prognostictools.es/neoBreast/inicio.aspx) to make individual predictions. The posterior probability of decline with mastectomy clearly differs based on age (e.g., the older and younger women suffer greater detriment). Alternative models (e.g., detailing chemotherapy regimens, adding anthracyclines or different nonlinear effects, etc.) were explored but did not fit the data-generating process much better.

**Table 2:** Odds ratios of the constrained partial proportional odds model.

	Health status thresholds	OR (95% HPDI)	Posterior probability of harm (OR < 1)
EORTC QLQ-C30 sum score, 60 vs 85	≥50	0.54 (0.27–1.12)	95%
	≥66	0.34 (0.20–0.62)	100%
	≥75	0.27 (0.15–0.47)	100%
	≥83	0.21 (0.11–0.38)	100%
Perceived risk of recurrence, very high vs medium-low	Any	0.47 (0.23–1.04)	97%
Perceived risk of recurrence, very high vs high	Any	0.84 (0.49–1.46)	73%
Perceived risk of recurrence, high vs medium-low	Any	0.56 (0.27–1.11)	56%
No ALND	Any	0.77 (0.40–1.52)	79%
Age, 40 vs 55 years	≥50	0.26 (0.08–0.82)	99%
	≥66	0.37 (0.15–0.92)	98%
	≥75	0.45 (0.19–10.6)	97%
	≥83	0.53 (0.21–1.28)	92%
TNM stage, 3 vs 2	Any	0.67 (0.27–1.68)	81%
TNM stage, 2 vs 1	Any	1.11 (0.63–2.01)	35%
Nontaxane regimen	Any	1.04 (0.55–1.84)	44%
Mastectomy	≥50	0.30 (0.11–0.84)	99%
	≥66	0.42 (0.20–0.91)	99%
	≥75	0.49 (0.24–0.99)	98%
	≥83	0.57 (0.28–1.23)	93%

We can see that the CPPO model assumes proportional odds for all variables, except for baseline QoL and age (that interacts with surgery). The model imposes a linear restriction on the coefficients of these variables that do not meet the proportional odds assumption with respect to cutoffs. In contrast, the variables that do meet the proportional odds assumption have a single odds ratio that is valid for the entire range of possible cutoffs. The EORTC QLQ-C30 sum score was measured before adjuvant chemotherapy.

*ALND* axillary lymph node dissection, *CI* confidence interval, *TNM* tumor–node–metastases, *OR* odds ratio, *HPDI* highest posterior density interval.

## Discussion

In this study, we have fit a CPPO model to probe the factors that predict global health status in women with breast cancer who undergo surgery and complete systemic adjuvant chemotherapy. This model was put forth by Peterson & Harrell in 1990 to model ordinal endpoints, easing the PO assumption, often unmet in analysis^15^. These predictions are tricky, since they entail a trade-off between parsimony and adequate model fit (e.g., the need to broaden parameters involves increased random error). Among the compromise solutions, the model used here imposes a linear constriction on certain selected covariates, which preserves simplicity^15^. Despite being deemed a valuable method, the absence of software has prevented it from being more widely used^12,21^. A package recently written to examine ordinal endpoints using this approach in COVID-19 trials affords us an opportunity for studies on QoL in cancer patients^22,23^.

As previously reported in the literature, the CPPO model confirms worse health status following total mastectomy vs BSC^10^, although it contributes nuances and additional insights. Firstly, while total mastectomy increases the probability of QoL scores in the lowest range, conserving the breast does not increase the odds of higher scores to the same degree, which must be taken into account when making predictions. Secondly, age is a predictive factor per se, albeit it is also a modifier of the effect of surgery. This interaction is clearly nonlinear, such that both extremes (younger and older women) fare worse. Nonlinear effects are adequately captured here using a spline (a function defined piecewise by polynomials). Thirdly, long-term predictions require that the starting point be taken into account, which cannot be done with bivariate methods. The baseline EORTC QLQ-C30 sum score is therefore germane and, likewise, nonlinear. This is in line with other data that point toward the existence of different trajectories in the evolution of QoL, depending on specific clinical profiles^24^.

The initial perception of risk of recurrence modulates the evolution of health status at completion of systemic therapy, in keeping with other series^5,25^. Regardless of evaluating participants’ perception of risk after their appointment with their oncologist, the correlation with TNM stage is discrete, given that women measure it similarly when facing stages I and II. The same holds true for the type of surgery. This indicates that after being informed, some women perceive a level of risk typically deemed ‘low’ compared to advanced stages or other neoplasms, as being ‘high’. A plausible explanation is that the use of adjuvant chemotherapy as an eligibility criterion in this study may have led many patients to consider that their risk, even with stage I, exceeds their actual probability of relapse. This perception is far from trivial, as it is projected on health status for at least the following six months and, therefore, has an identifiable clinical impact. Nonetheless, the reader must be cognizant of the subtlety that the woman was asked with respect to her subjective perception and not about her quantitative prognosis. Be that as it may, it is not clear that there is a component of misunderstanding or insufficient explanation.

Fourthly, the Bayesian formulation enables gradual results and actionable probabilities to be obtained. Thus, constrained by the small sample size, the evidence is limited for ALND or TNM stage III tumors, although the model suggests that a possible negative effect of these factors is highly probable. Depending on the external context, clinicians must determine the degree to which the differences in probability are clinically relevant in each case. This makes the Bayesian approach an appealing, pragmatic method. In this line, Spiegelhalter et al. have proposed that the post hoc Bayesian interpretation be routinely used in clinical trial analyses^26^. The Bayesian CPPO model brings together the requirements to meet this function in terms of interpreting gradual QoL outcomes. Fifthly, in addition to causal inference, the complete Bayesian model can be used for patient profile-based predictions. Thus, the tool enables nomograms or online calculators to be built, factoring in the aforementioned considerations. This allows clinical questions to be answered, such as: What is my probability of achieving a certain level of QoL according to my specific characteristics? On the other hand, the bivariate methods so assiduously applied, are unable to capture these nuances, or do so only partially^10^.

The web calculator is potentially useful, and its relevance must be put into context. BCS was developed more than 40 years ago as an alternative to modified radical mastectomy (MRM) in T1-2 tumors^27,28^. Survival following BCS and radiotherapy is comparable to survival following MRM^29,30^, with better esthetic outcomes. Over time, the indication of BCS was extended to T3 tumors or tumors with node involvement (N + ), after responding to neoadjuvant chemotherapy^27,31^. Given that survival outcomes with BCS and MRM are commensurate when BSC is indicated, knowing the impact on mid-term QoL acquires special interest in decision making^4^. Several studies have reported differences in specific QoL domains related to type of surgery^32–36^. A recent meta-analysis confirmed that BCS improved certain aspects of QoL (e.g., body image, future perspectives, or treatment sequelae), but not all of them^10^, requiring more prospective studies to elucidate the impact^37^. After half a century of clinical research in this field, it is striking that important aspects such as the modification of the effect on QoL according to age, or individualized prediction, are still seldom explored^10^.

Our study has various limitations. First of all, the specific EORTC module for women with breast cancer, the QLQ-BR23, was not administered^38^. The use of the global health status detects generic changes and accounts for three quarters of the variability of the complete score, but may be insensitive to certain aspects. Secondly, QoL is a dynamic construct that varies based on time since interventions. However, some trajectories are stable according to individual clinical profile^24^. Here, the response variable was the health status evaluated at the end of systemic chemotherapy since this is the only post-baseline timepoint contemplated. The QoL recorded prior to chemotherapy was used as a covariate (baseline measurement taken as the starting point). However, it is plausible that the progressive disappearance of adverse effects would accentuate the differences between BCS and mastectomy at a later stage of evolution (for instance, 2 years). Therefore, assessing QoL at several timepoints, over a minimum of 1–2 years of follow-up might be more relevant. If more measures were available, the CPPO model allows random effects from longitudinal data to be integrated. Moreover, the reader should be aware that baseline QoL scores were obtained post surgery and may be worse than pre-surgery scores. As in other studies, NEOCOPING has questionnaires that were not completed at the 6-month timepoint. Nonetheless, the clinical scenario is adjuvancy, and most are confirmed missing at random and not due to clinical decline. In any case, the library makes it possible to work with multiple imputations of these data, if necessary^22^. Thirdly, other clinical elements that can affect QoL, such as early breast reconstruction or radiotherapy, have not been completed. These factors do indeed modulate QoL in some patients. Other uncommon variables or those not supported by the model, such as level of education, occupation, marital status, or specific comorbidities, might also be relevant for some patients. Fourth, the model was causally specified to explore the effect of surgery; consequently, the interpretation of the remaining effect estimates, including ‘secondary exposures’ such as perceived risk of recurrence, can be challenging^39^. Fifth, the self-report subjective measures may have limitations, such as response bias (social desirability, inaccurate memory, etc.) and difficulty in fully comprehending the questionnaire. Finally, the model must be independently and prospectively validated for its clinical implementation.

Bearing these limitations in mind, the conditions of applicability must always be verified. Consequently, the conclusions are generalizable to women who have undergone surgery for breast cancer for whom adjuvant chemotherapy initiation is being contemplated. Despite the fact that this limits the sphere of application of the model and the gradual decrease in chemotherapy notwithstanding thanks to predictive genomic tests, chemotherapy continues to be part of many women’s experience of cancer (e.g., luminal breast cancer in premenopausal or postmenopausal women with positive nodes and high-risk negative nodes, as well as in most triple-negative or HER2-positive tumors)^40^. One could speculate that the differences between mastectomy and BCS might be different in women without chemotherapy, given that the impact of surgical sequelae and changes in self-image would not have been diluted by the full burden of post-chemotherapy side effects.

As for the generalizability of the method itself, the Bayesian CPPO model comprises a new, extremely versatile, and flexible tool to investigate QoL associated with cancer in multiple scenarios, facilitating the obtention of rich, individualized descriptions of patients’ evolution.

In short, we have fit a Bayesian ordinal model using software programmed for COVID-19 trials to illustrate its usefulness in analyzing QoL in oncological patients. The model makes it possible to overcome certain issues associated with QoL analyses with assumable complexity and accurately capture the main factors that affect global health status (type of surgery, interaction with age, or perceived risk of recurrence). The study demonstrates the feasibility of post hoc Bayesian analysis with QoL data that can be implemented in clinical trials. This Bayesian model is also a potentially useful tool in making decisions grounded in the foreseeable evolution of QoL, when facing therapies of comparable benefit.

## Methods

### Patients and study design

The data are from a prospective cohort of individuals with early and locally advanced breast cancer from the NEOCOPING multicohort study. This study was promoted by the Continuous Care Group of the Spanish Society of Medical Oncology (SEOM, for its acronym in Spanish) and was conducted at 17 Spanish hospitals between 2016 and 2019 (Supplementary Table 1)^41–45^. The participating centers are tertiary university hospitals distributed all over Spain. The participants had undergone surgery with curative intent for nonadvanced cancers for which clinical guidelines report adjuvant chemotherapy as a valid alternative. The study sought to explore biopsychosocial and pathological aspects that affected the quality of life, coping, and oncologist–patient communication.

For this analysis, women ≥18 years were chosen, with a histologically confirmed diagnosis of breast cancer, stages TNM I–III, and indication of adjuvant chemotherapy. All participants were recruited during the interval between surgery and initiation of adjuvant chemotherapy. The patients included were those who consulted with Medical Oncology and were selected consecutively and prospectively by the medical oncologist. Exclusion criteria included receiving neoadjuvant therapy and scheduled to receive adjuvant hormone therapy alone or radiotherapy without chemotherapy.

#### Ethical statement

This study was performed in accordance with the ethical standards of the Declaration of Helsinki and its subsequent amendments. This observational, noninterventional trial was approved by the Research Ethics Committee of the Principality of Asturias (19 January 2015) and by the Spanish Agency of Medicines and Medical Devices (AEMPS) (number: L34LM-MM2GH-Y925U-RJDHQ).

#### Informed consent statement

All subjects signed informed consent forms and agreed to participate prior to completing baseline questionnaires.

#### Consent for publication

Informed consent and approval by the competent national authorities includes permission for publication and dissemination of the data.

### Measures and variables

Participants completed the self-report European Organisation for Research and Treatment of Cancer Quality of Life Core Questionnaire (EORTC QLQ-C30) scale^46^. This instrument can be downloaded in English and Spanish at https://qol.eortc.org/. This questionnaire is a cancer-specific QOL instrument, psychometrically validated for most tumors, and the most widely used tool to quantify cancer patients’ QoL^38,47,48^. It is a 30-item, self-report questionnaire that covers five QoL dimensions: physical (5 items), role (2), emotional (4), social (2), and cognitive (2); an overall health status assessment (2), and the following specific symptoms: pain (2), fatigue (3), and nausea and vomiting (2 items). It contains six items to appraise financial impact, as well as other symptoms (such as constipation, sleep problems, hyporexia, etc.). The score is expressed on a scale of 0 to 100 (the higher the score, the better the QoL or more symptoms according to the item)^49^ and takes less than 15 min to complete^50^. The reliability of these scales denotes suitable internal consistency (Cronbach’s alpha ≈0.67–0.92)^51^. The information from the questionnaire was agglutinated into a sum score and evaluated in line with the scoring manual recommended by the EORTC QoL Group^49,52^. The scale has been translated and validated in Spanish^53^.

Since the questionnaire has items that are prima facie scantly applicable to subjects with few symptoms (e.g., diarrhea, nausea, fatigue…), the calculator uses the global health status (average of items 29 and 30) at 6 months as the response variable. Health status correlated closely to the EORTC sum score (Spearman, *ρ* = 0.88) and accounted for 75% of its variability by means of only two items: “How would you rate your overall health during the past week?” and “How would you rate your overall quality of life during the past week?”.

When selecting covariates, the limiting sample size was contemplated to evaluate an ordinal response variable, as well as the need to nonlinearly model some variables^54^. Taking into account the maximum number of parameters the model can support, the covariates were selected after a bibliographic review and at the researchers’ discretion. These predictors were age (continuous, nonlinear), TNM classification of malignant tumors, 8th edition (stage I, II, III), patient’s perceived risk of recurrence (4-point Likert scale: low/intermediate/high/very high risk), type of surgery on the primary tumor (total mastectomy vs BCS), axillary surgery (axillary lymph node dissection (ALND) [yes vs no]), the pre-chemotherapy EORTC QLQ-C30 sum score, and planned chemotherapy regimen (taxane-containing regimens and use of anthracyclines). Other clinical or sociodemographic variables (e.g., social status, number of children, etc.) were considered for descriptive purposes.

### Procedures

The QoL evaluations were performed twice, once after the first appointment with the oncologist, approximately 1 month following surgery for the primary tumor and 1 week prior to initiating adjuvant therapy, and again, during the month following completion of adjuvant chemotherapy, some 6 months after initiation and prior to undertaking adjuvant radiotherapy/endocrine therapy when necessary. Baseline questionnaires were completed by the participants themselves after their visit to the oncologist, during which they were informed about the risk of relapse and indication of adjuvant therapy.

### Statistical analysis

The rmsb package enables Bayesian CPPO models to be fitted. Vague priors (noninformative) were used here for coefficients. As the response variable, the health status endpoint is an ordinal variable with 13 levels, scaled from 0-100. The CPPO model determines the probability that the participants would have a health status *Y* ≥ *j*, with *j* being each of the 13 levels. The PO assumption was assessed separately for each predictor computing logits for each proportion of the form *Y* ≥ *j*, with *j* being the thresholds for the ordinal response variable. When the PO is maintained, the difference of logits between various *j* values should be similar for different levels of the predictor^54^. Bearing this in mind, we fitted a CPPO model as per the formulation proposed by Peterson & Harrell^15^. Here, this model assumes PO for all variables, except for age and baseline EORTC QLQ-C30 sum score. Nonlinear relations were assessed by means of restricted cubic splines (age) or adding quadratic terms to the equation (baseline EORTC QLQ-C30 summary score). Moreover, the model contemplates the interaction between surgery and age. We run a Markov chain Monte Carlo (MCMC) method with 4 chains, 2000 iterations, and a burn-in of 1000 samples, in each one. The c-index and the Brier score have been used as measures of model performance. For comparison between alternative models (e.g., with or without the covariate ‘anthracyclines’), we used the widely applicable information criterion (WAIC) for parsimony^55^. Odds ratios (OR) < 1 indicate worse QoL scores in the presence of a binary variable. In the variables that deviate from the PO assumption, the model imposes a linear restriction on the coefficients (e.g., the variable has a different OR for each QoL score cutoff, but all of them are linearly related). In contrast, those variables that do meet the proportional odds assumption have a single odds ratio that is applicable to the entire range of possible cutoffs. To illustrate these concepts, the prediction has been visually depicted for the different levels, odds ratios, and partial effects, and it has been contrasted with the evaluation of average effects stratified for a single variable. The global health status predictive model was depicted graphically by a web calculator programmed in .NET and R.

For comparative purposes, a frequentist PO model and a multinomial model were also fitted. QoL scores according to type of surgery were also compared by means of two-samples Wilcoxon tests (*α* = 0.05, two-tailed tests).

### Reporting summary

Further information on research design is available in the Nature Research Reporting Summary linked to this article.

## Supplementary information

Supplementary Information

Reporting Summary

## Data Availability

The data generated and analyzed during this study are described in the following data record: 10.6084/m9.figshare.14681274^56^. The breast cancer quality of life datasets are openly available as part of the data record in the files ‘breast_cppo.xlsx’ (underlying Figs. 2–4, Tables 1–2, Supp Figs. 2–8, Supp Tab 2 of the related article), ‘breast_scores.xlsx’ (underlying Fig. 1) and ‘breast_qlq_c30.xlsx’ (underlying Supp Tab 3).

## Code availability

All analyses were performed with the R version 4.0.2 software^57^, including the rmsb package^58,59^. The R Code is available upon request to the authors.
