# Supplementary material for: Prediction of quality of life in early breast cancer upon completion of adjuvant chemotherapy
Source: NPJ Breast Cancer. 2021 Jul 13;7:92. doi: 10.1038/s41523-021-00296-8 (PMC8277774; doi:10.1038/s41523-021-00296-8)
Supplement: Supplementary file 1 — Supplementary Information [file 41523_2021_296_MOESM1_ESM.pdf]

Supplementary Figure 1. Flowchart of patients in the study

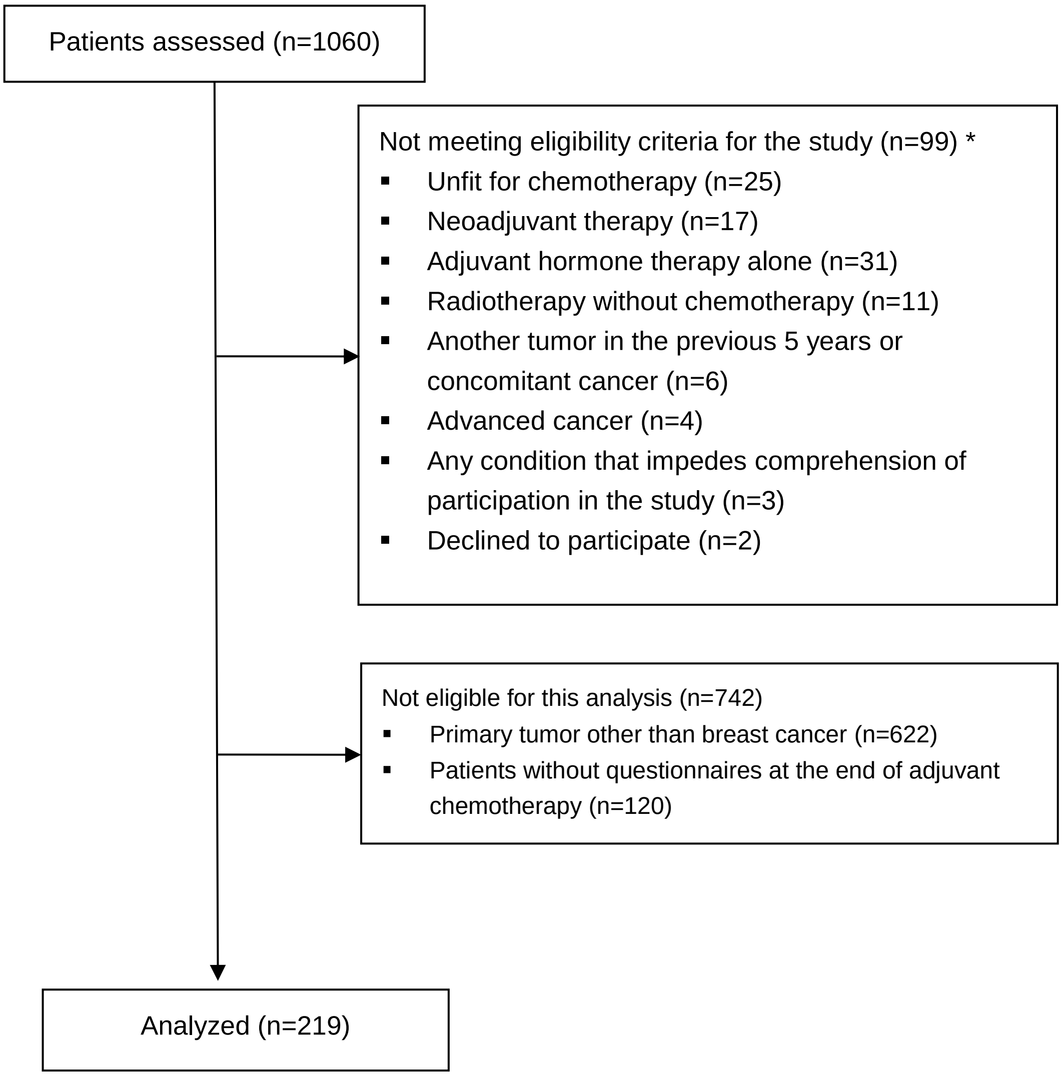

Supplementary Figure 2. Distribution of global health status scores

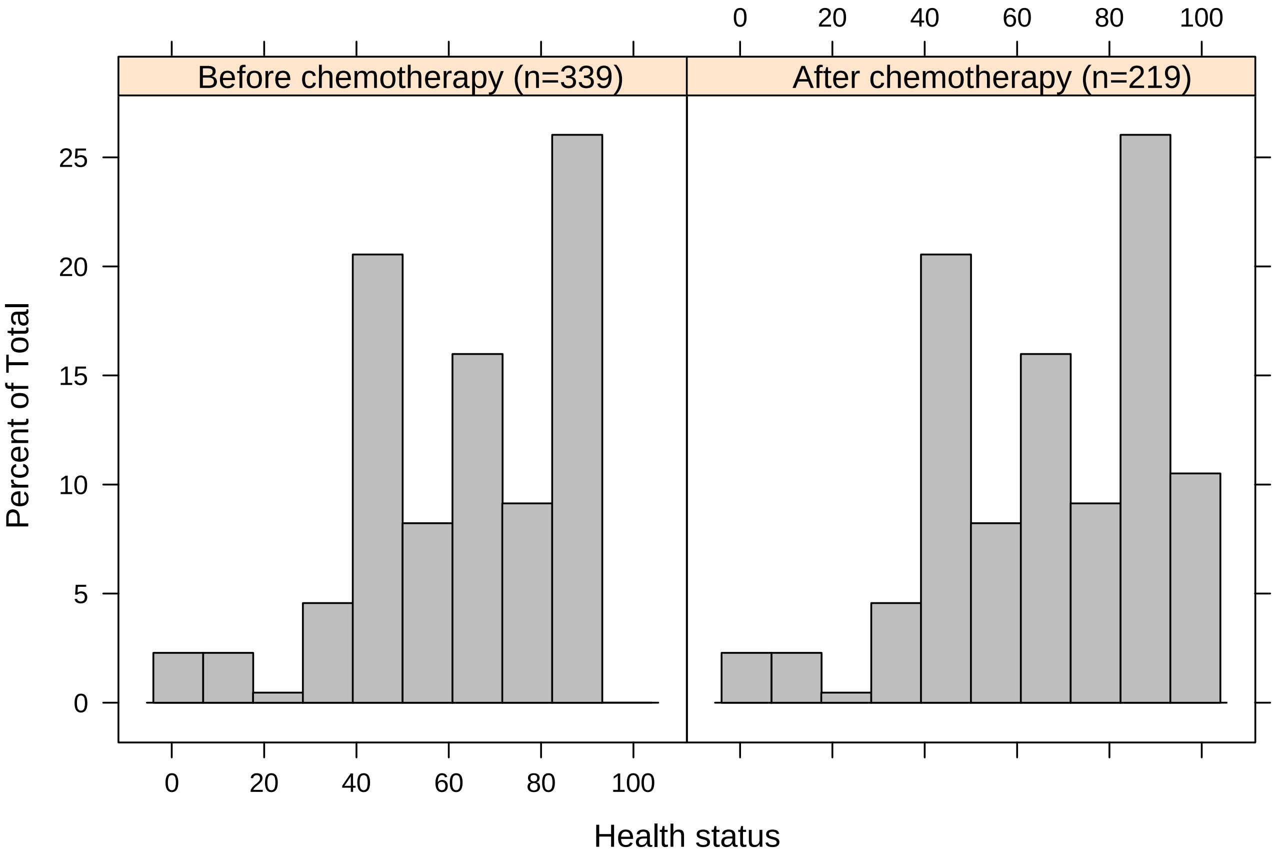

Supplementary Figure 3A. Perceived risk of recurrence based on stage

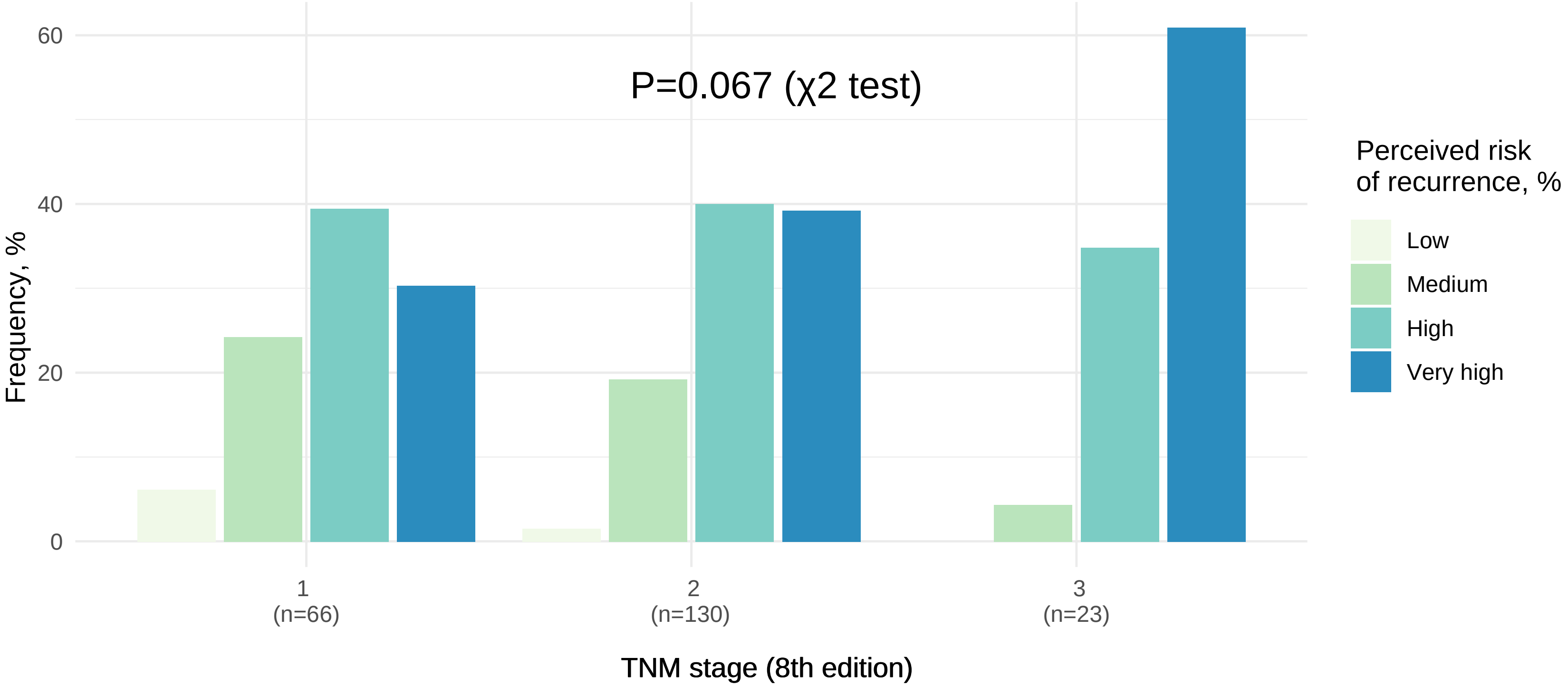

Note: The p-value is established by means of a χ2 test

Supplementary Figure 3B. Perceived risk of recurrence based on surgery

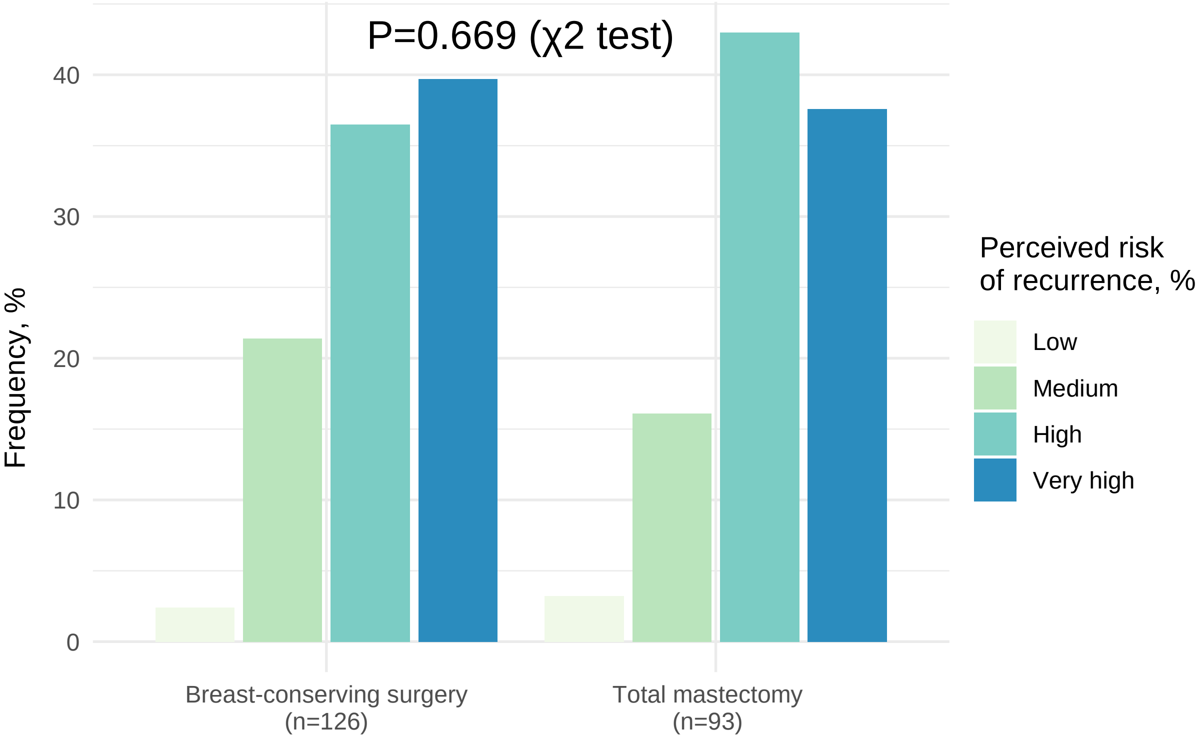

Note: The p-value is established by means of a χ2 test

Supplementary Figure 4. Proportional odds model (frequentist)

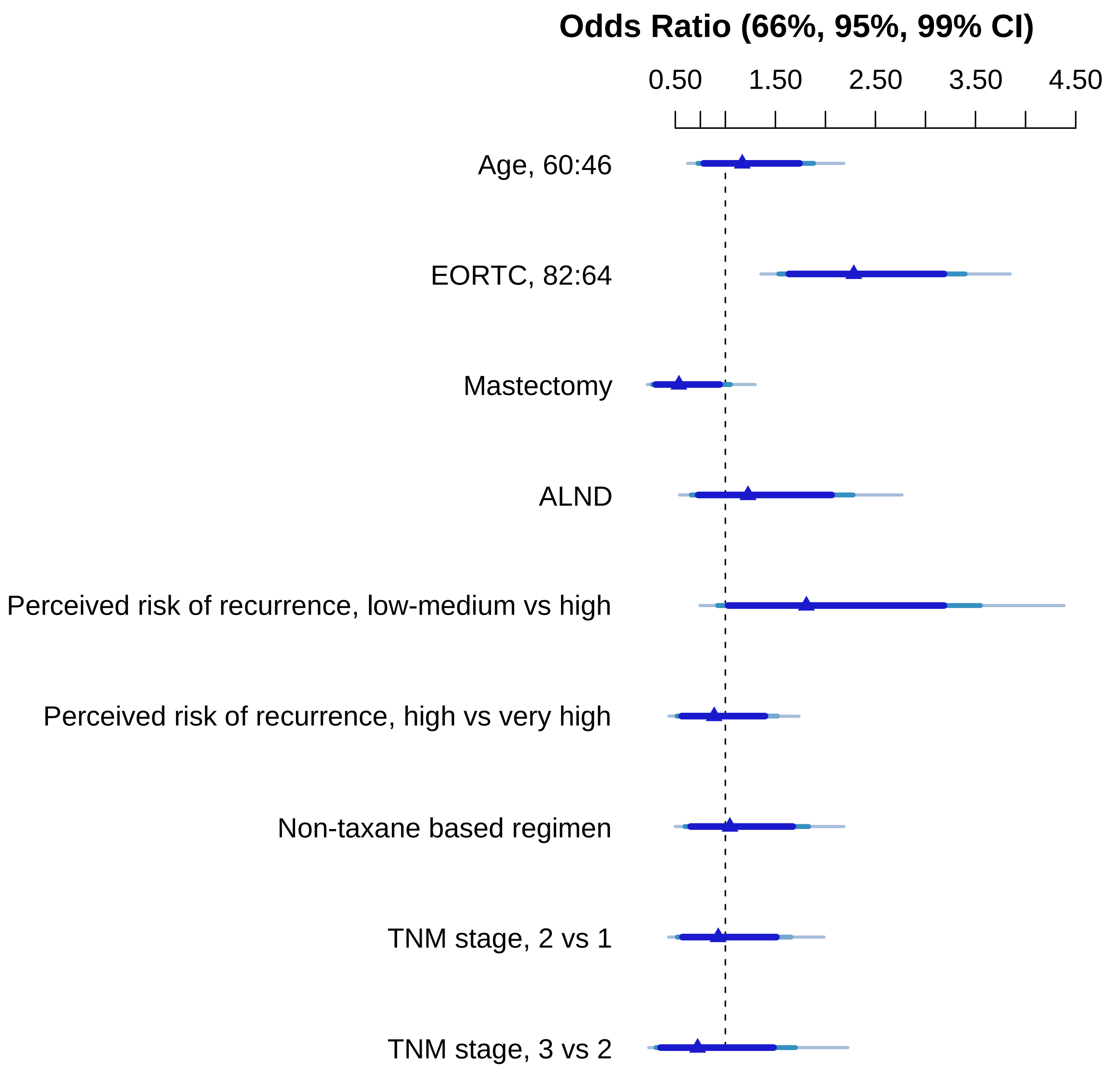

Abbreviations: ALND= axillary lymph node dissection; EORTC= European Organisation for Research and Treatment of Cancer; TNM= tumor-node-metastases; CI= confidence interval. Note: The EORTC variable refers to the sum score before chemotherapy. The response variable (endpoint) is the health status after chemotherapy.

Supplementary Figure 5. Separate evaluations of the proportional odds (PO) assumption for each of the model's covariates.

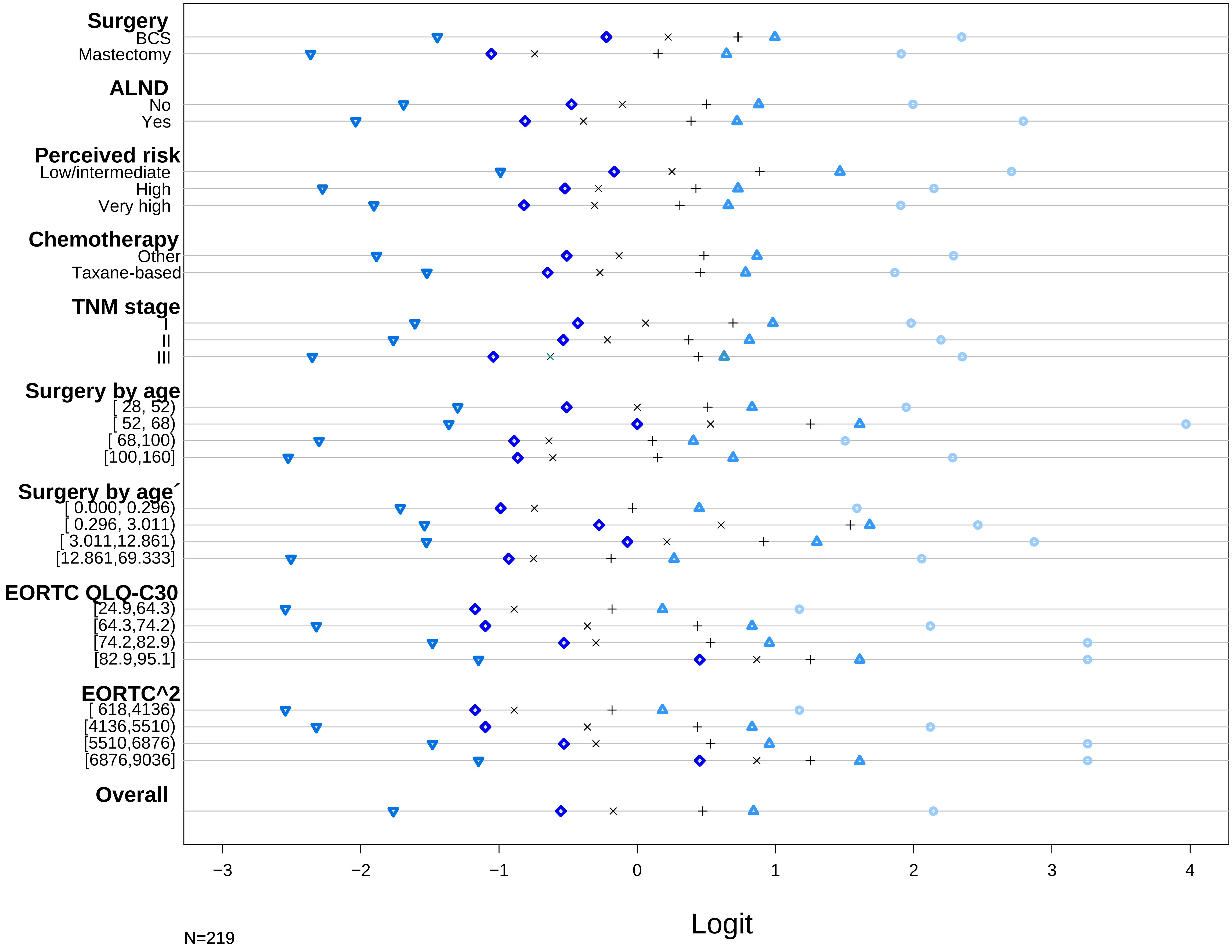

Note: In this case, circle, triangle, plus sign, cross, diamond, and inverted triangle correspond to the endpoint level of Y ≥ 50, 58, 66, 75, 83, and 91, respectively. The PO is checked by inspecting the vertical homogeneity of the distance of any of these symbols within the levels for each predictor. The response variable (endpoint) is the health status after chemotherapy. Abbreviations: BSC= breast-conserving surgery; ALND= axillary lymph node dissection; TNM= tumor-node-metastases; EORTC= European Organisation for Research and Treatment of Cancer. The EORTC variable refers to the sum score before chemotherapy.

Supplementary Figure 6. Multinomial (frequentist) model.

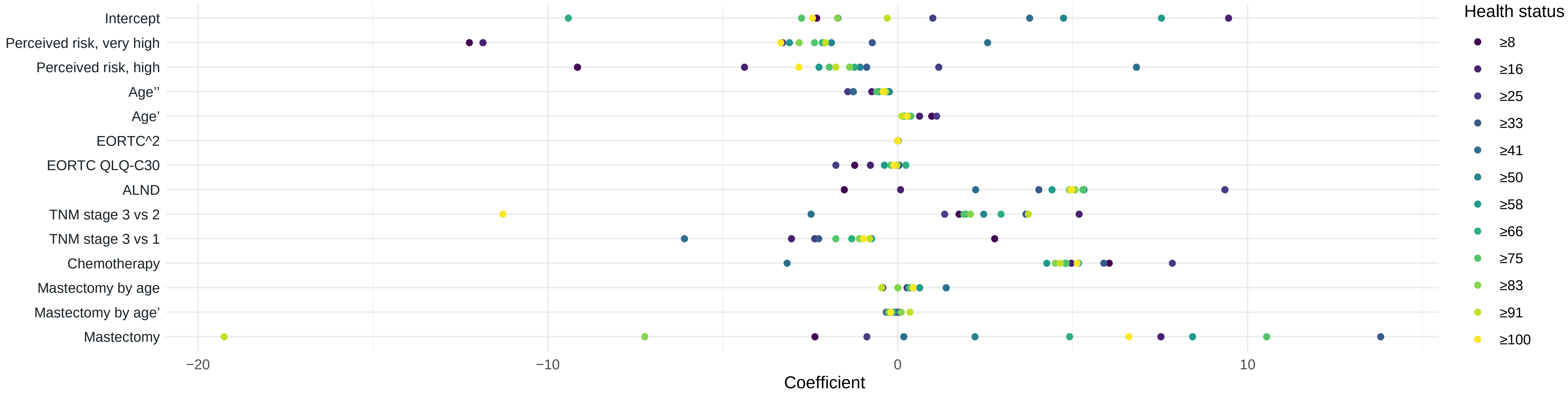

Each individual point represents one of the model's parameters. The reader can see the point to which the model is further complicated after assuming multinomial effects (one coefficient for each cut-off of the endpoint as illustrated by the color gradient).

Abbreviations: ALND, axillary lymph node dissection; TNM= tumor-node-metastases; EORTC= European Organisation for Research and Treatment of Cancer.

The response variable (endpoint) is the health status after chemotherapy. The EORTC variable refers to the sum score after chemotherapy.

Supplementary Figure 7: Coefficients of the constrained partial proportional odds model.

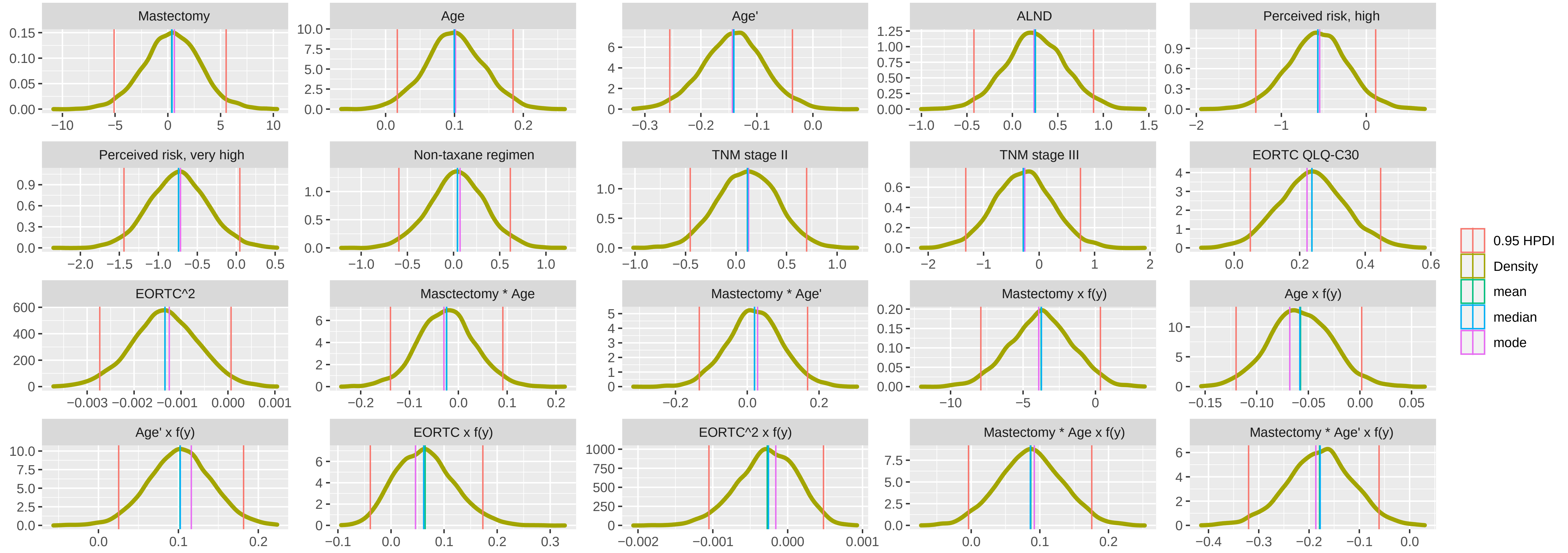

Abbreviations: ALND, axillary lymph node dissection; TNM= tumor-node-metastases; OR= odds ratio; HPDI= highest posterior density interval; EORTC= European Organisation for Research and Treatment of Cancer. Note: The EORTC variable refers to the sum score before chemotherapy.
The response variable is the health status after chemotherapy.

Supplementary Figure 8. Model performance measures.

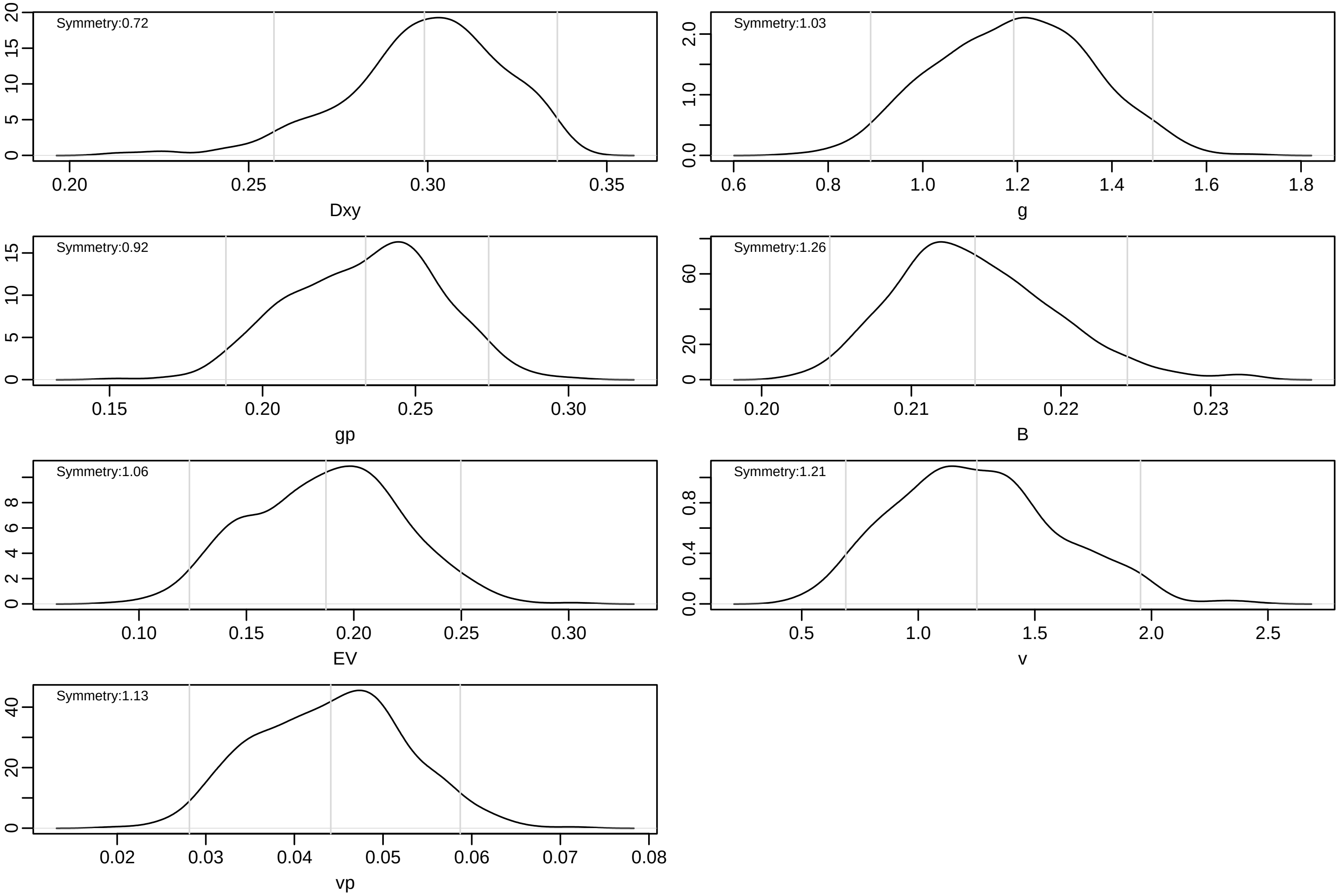

Dxy= Somers' Dxy rank correlation between predicted and observed. The concordance probability (c-index; AUROC in the binary Y case) can be obtained from the relationship Dxy=2(c0.5); g= Gini's mean difference: the average absolute difference over all pairs of linear predictor values; gp= Gini's mean difference on the predicted probability scale; B= Brier score; EV= explained variation; v= variance of linear predictor; vp= variable of estimated probabilities.

Supplementary Table 1: Hospitals participating in the study

Hospital Virgen de la Macarena, Sevilla
Hospital Quirón Salud Sagrado Corazón, Sevilla
Hospital Universitario Central de Asturias, Oviedo
Hospital Universitario Son Espases, Mallorca
Hospital Universitario Insular de Gran Canarias, Las Palmas
Hospital Universitario de Canarias, Tenerife
Hospital Universitario Marqués de Valdecilla, Santander
Hospital General Virgen de la Luz, Cuenca
Hospital General de Segovia, Segovia
Hospital Universitari Santa Creu i Sant Pau, Barcelona
Hospital Universitario La Paz, Madrid
Hospital Universitario La Princesa, Madrid
Hospital Universitario Fundación Alcorcón, Madrid
Hospital Universitario del Sureste, Arganda del Rey, Madrid
Hospital Universitario Morales Meseguer, Murcia
Hospital Galdakao-Usansolo, Galdacano, Vizcaya
Hospital General Universitario de Elche, Elche

Supplementary Table 2. Characteristics of patients according to type of surgery (N=219)

	BSC N=126 (%)	Total mastectomy N=93 (%)
**Age (median, range)**	53 (32-76)	52 (28-80)
**Axillary lymph node dissection**	21 (16.7)	31 (33.3)
**TNM stage**		
**I**	46 (36.5)	20 (21.5)
**II**	69 (54.8)	61 (65.6)
**III**	11 (8.7)	12 (12.9)
**Chemotherapy regimen**		
**Taxane-based**	89 (70.6)	63 (67.7)
**Other**	37 (29.4)	30 (32.3)
**HER2-positive cancer**	39 (31.0)	14 (15.1)
**ECOG Performance status**		
**0**	95 (75.4)	70 (75.3)
**1**	29 (23.0)	21 (22.6)
**2**	2 (1.6)	2 (2.2)
**Social status**		
**Secondary or higher education**	65 (51.6)	50 (53.8)
**Employed**	64 (50.8)	36 (38.7)
**Married/ partnered**	96 (76.2)	74 (79.6)
**Number of children**		
**None**	27 (21.4)	14 (15.2)
**1**	20 (15.9)	20 (21.7)
**2**	53 (42.1)	48 (52.2)
**>2**	26 (20.6)	10 (10.9)

Abbreviations: BSC, breast-conserving surgery; MRM, modified radical mastectomy.

Supplementary Table 3. Assessment of quality of life through the EORTC QLQ-C30 questionnaire

	**Overall (mean, sd)**		**BCS (mean, sd)**		**Total mastectomy (mean, sd)**		**p-value**	
	**Baseline**	**At the end of Ct**	**Baseline**	**At the end of Ct**	**Baseline**	**At the end of Ct**	**Baseline**	**At the end of Ct**
**Global health status**	70.4 (18.6)	67.0 (22.3)	71.5 (18.3)	70.6 (21.7)	69.3 (18.9)	62.0 (22.6)	0.238	**0.004**
**Physical funct.**	86.2 (14.0)	80.8 (17.7)	88.6 (12.8)	83.1 (16.6)	84.6 (15.0)	77.7 (18.7)	**0.007**	**0.024**
**Role funct.**	75.6 (25.7)	71.6 (28.9)	77.9 (23.6)	77.0 (27.8)	74.1 (27.9)	64.4 (28.8)	0.629	**<0.001**
**Emotional funct.**	68.9 (26.6)	72.3 (26.7)	68.7 (25.8)	76.4 (24.3)	69.1 (27.5)	66.9 (28.8)	0.680	**0.001**
**Social funct.**	74.6 (25.7)	72.2 (28.5)	76.6 (24.9)	75.1 (27.9)	72.5 (26.4)	68.5 (29.1)	0.158	0.060
**Cognitive funct.**	83.0 (21.2)	80.0 (24.4)	82.5 (20.8)	81.8 (23.6)	83.2 (21.6)	77.5 (25.3)	0.705	0.146
**Fatigue**	30.6 (25.7)	43.1 (28.0)	31.4 (26.2)	38.1 (26.3)	29.8 (24.2)	49.5 (28.5)	0.689	**0.002**
**Nausea&vomiting**	9.5 (18.2)	9.8 (18.9)	9.9 (18.3)	7.2 (18.0)	9.0 (17.8)	12.4 (19.5)	0.593	**0.005**
**Pain**	20.6 (25.5)	25.2 (28.4)	18.8 (24.3)	22.1 (25.9)	22.5 (26.8)	28.3 (30.5)	0.249	0.217
**Dyspnoea**	6.2 (5.5)	9.8 (23.6)	6.7 (19.1)	8.0 (20.6)	5.5 (18.1)	11.6 (26.2)	0.382	0.315
**Insomnia**	35.0 (33.5)	36.2 (33.5)	33.7 (33.7)	33.6 (32.7)	36.5 (33.2)	38.8 (34.3)	0.376	0.246
**Appetite loss**	19.5 (28.5)	17.6 (27.7)	19.1 (28.4)	13.7 (24.1)	19.9 (28.8)	22.7 (31.3)	0.798	0.022
**Constipation**	23.9 (33.2)	26.1 (31.6)	25.2 (33.4)	25.5 (31.6)	22.4 (33.0)	26.6 (31.7)	0.296	0.754
**Diarrhea**	13.0 (24.0)	13.2 (23.7)	12.3 (22.9)	10.4 (20.3)	13.7 (25.1)	17.0 (27.1)	0.867	0.063
**Financial difficulties**	18.1 (28.3)	14.4 (26.4)	16.1 (26.3)	11.4 (23.0)	20.2 (30.2)	18.3 (29.9)	0.335	0.095
**Symptoms score**	19.6 (16.0)	21.7 (16.5)	19.4 (15.8)	19.0 (15.3)	19.9 (16.2)	24.4 (17.2)	0.765	0.002

Abbreviatures: BSC, breast-conserving surgery; sd, standard deviation; Ct, adjuvant chemotherapy; funct., functioning. The term 'baseline' refers to the QoL scores prior to chemotherapy. The p-values proceed from two-sample Wilcoxon tests that compare the distribution of scores in subjects with BSC vs total mastectomy. This evaluation is performed separately before and after chemotherapy
