# Supplementary material for: Prediction of quality of life in early breast cancer upon completion of adjuvant chemotherapy
Source: NPJ Breast Cancer. 2021 Jul 13;7:92. doi: 10.1038/s41523-021-00296-8 (PMC8277774; doi:10.1038/s41523-021-00296-8)
Supplement: Supplementary file 2 — Reporting Summary [file 41523_2021_296_MOESM2_ESM.pdf]

nature research

Corresponding author(s): Alberto Carmona-Bayonas

Last updated by author(s): Apr 7, 2021

Reporting Summary

Nature Research wishes to improve the reproducibility of the work that we publish. This form provides structure for consistency and transparency in reporting. For further information on Nature Research policies, see our Editorial Policies and the Editorial Policy Checklist.

Statistics

For all statistical analyses, confirm that the following items are present in the figure legend, table legend, main text, or Methods section.

n/a	Confirmed	
☐	☒	The exact sample size (*n*) for each experimental group/condition, given as a discrete number and unit of measurement
☐	☒	A statement on whether measurements were taken from distinct samples or whether the same sample was measured repeatedly
☐	☒	The statistical test(s) used AND whether they are one- or two-sided<br>*Only common tests should be described solely by name; describe more complex techniques in the Methods section.*
☐	☒	A description of all covariates tested
☐	☒	A description of any assumptions or corrections, such as tests of normality and adjustment for multiple comparisons
☐	☒	A full description of the statistical parameters including central tendency (e.g. means) or other basic estimates (e.g. regression coefficient) AND variation (e.g. standard deviation) or associated estimates of uncertainty (e.g. confidence intervals)
☐	☒	For null hypothesis testing, the test statistic (e.g. *F*, *t*, *r*) with confidence intervals, effect sizes, degrees of freedom and *P* value noted<br>*Give P values as exact values whenever suitable.*
☐	☒	For Bayesian analysis, information on the choice of priors and Markov chain Monte Carlo settings
☒	☐	For hierarchical and complex designs, identification of the appropriate level for tests and full reporting of outcomes
☒	☐	Estimates of effect sizes (e.g. Cohen's *d*, Pearson's *r*), indicating how they were calculated

Our web collection on statistics for biologists contains articles on many of the points above.

Software and code

Policy information about availability of computer code

Data collection	Data were collected through a web platform (http://www.neocoping.es/) programmed in .NET.
Data analysis	All analyses were performed with the R version 4.0.2 software

For manuscripts utilizing custom algorithms or software that are central to the research but not yet described in published literature, software must be made available to editors and reviewers. We strongly encourage code deposition in a community repository (e.g. GitHub). See the Nature Research guidelines for submitting code & software for further information.

Data

Policy information about availability of data

All manuscripts must include a data availability statement. This statement should provide the following information, where applicable:

- Accession codes, unique identifiers, or web links for publicly available datasets
- A list of figures that have associated raw data
- A description of any restrictions on data availability

The data generated and analyzed during this study are available upon request.

Field-specific reporting

Please select the one below that is the best fit for your research. If you are not sure, read the appropriate sections before making your selection.

☒ Life sciences ☐ Behavioural & social sciences ☐ Ecological, evolutionary & environmental sciences

For a reference copy of the document with all sections, see nature.com/documents/nr-reporting-summary-flat.pdf

Life sciences study design

All studies must disclose on these points even when the disclosure is negative.

Sample size	The sample size was fixed, limited to the number of patients available in the database. Model building was then based on the limiting sample size of this study for an ordinal response variable.
Data exclusions	No data were filter here.
Replication	We repeated bayesian a frequentist versions of the models.
Randomization	Not applicable.
Blinding	Not applicable.

Reporting for specific materials, systems and methods

We require information from authors about some types of materials, experimental systems and methods used in many studies. Here, indicate whether each material, system or method listed is relevant to your study. If you are not sure if a list item applies to your research, read the appropriate section before selecting a response.

Materials & experimental systems

n/a	Involved in the study	
☒	☐	Antibodies
☒	☐	Eukaryotic cell lines
☒	☐	Palaeontology and archaeology
☒	☐	Animals and other organisms
☐	☒	Human research participants
☒	☐	Clinical data
☒	☐	Dual use research of concern

Methods

n/a	Involved in the study	
☒	☐	ChIP-seq
☒	☐	Flow cytometry
☒	☐	MRI-based neuroimaging

Human research participants

Policy information about studies involving human research participants

Population characteristics	The main covariates were age (continuous, non-linear), TNM classification of malignant tumors, 8th edition (stage I, II, III), patient's perceived risk of recurrence (4-point Likert scale: low/ intermediate/ high/ very high risk), type of surgery on the primary tumor (total mastectomy vs BCS), axillary surgery (axillary lymph node dissection (ALND) [yes vs no]), the pre-chemotherapy EORTC QLQ-C30 sum score, and planned chemotherapy regimen (taxane-containing regimens and use of anthracyclines). Other clinical or sociodemographic variables (e.g., social status, number of children, etc.) were considered for descriptive purposes.
Recruitment	The data are from a prospective cohort of individuals with early and locally advanced breast cancer from the NEOCOPING multicohort study. This study was promoted by the Continuous Care Group of the Spanish Society of Medical Oncology (SEOM, for its acronym in Spanish), and was conducted at 17 Spanish hospitals between 2016 and 2019. The participating centers are tertiary university hospitals distributed all over Spain. The participants had undergone surgery with curative intent for non-advanced cancers for which clinical guidelines report adjuvant chemotherapy as a valid alternative.<br>For this analysis, women ≥18 years were chosen, with a histologically confirmed diagnosis of breast cancer, stages TNM I-III, and indication of adjuvant chemotherapy. All participants were recruited during the interval between surgery and initiation of adjuvant chemotherapy. The patients included were those who consulted with Medical Oncology and were selected consecutively and prospectively by the medical oncologist. Exclusion criteria included receiving neoadjuvant therapy and scheduled to receive adjuvant hormone therapy alone or radiotherapy without chemotherapy.
Ethics oversight	The study was approved by the Research Ethics Committee of reference (Principality of Asturias, nº 10/15), the Research Ethics Committee of each participating hospital, and by the Spanish Agency of Medicines and Medical Devices (AEMPS)

(number L34LM-MM2GH-Y925U-RJDHQ). All subjects signed informed consent forms and agreed to participate prior to completing baseline questionnaires.

Note that full information on the approval of the study protocol must also be provided in the manuscript.
